# Establishing Sex-Dependent Reference Intervals for KL-6 in Danish Adults

**DOI:** 10.3390/diagnostics13111951

**Published:** 2023-06-02

**Authors:** Stine Bakkensen Bruun, Jeppe Buur Madsen, Claus Lohman Brasen

**Affiliations:** 1 Department of Biochemistry and Immunology, Lillebaelt Hospital, University Hospital of Southern Denmark, Beriderbakken 4, 7100 Vejle, Denmark; 2 Department of Regional Health Research, University of Southern Denmark, J. B. Winsløwsvej 19, 5000 Odense, Denmark

**Keywords:** Krebs von den Lungen-6 (KL-6), latex-particle-enhanced turbidimetric immunoassay, reference interval, normal values

## Abstract

Krebs von den Lungen-6 (KL-6) is a promising biomarker for the diagnosis and prognosis of interstitial lung disease. However, reference intervals in Northern Europeans remain to be established using a latex-particle-enhanced turbidimetric immunoassay. The participants were Danish blood donors subjected to strict health requirements. Analyses were performed using the Nanopia KL-6 reagent on the cobas 8000 module c502. Sex-partitioned reference intervals were determined using a parametric quantile approach according to the Clinical and Laboratory Standards Institute guideline EP28-A3c. The study included 240 participants—121 females and 119 males. The common reference interval was 59.4–398.5 U/mL (95% confidence intervals (CI) for the lower and upper limits were 47.3–71.9 and 369.5–430.1, respectively). For females, the reference interval was 56.8–324.0 U/mL (95% CIs for the lower and upper limits were 36.1–77.6 and 303.3–344.7, respectively). For males, the reference interval was 51.5–448.7 U/mL (95% CIs for the lower and upper limits were 32.8–71.2 and 397.3–508.1, respectively). These results emphasize the importance of sex partitioning when evaluating KL-6 reference intervals. The reference intervals increase the clinical applicability of the KL-6 biomarker and provide a basis for future scientific studies of its utility in patient management.

## 1. Introduction

Krebs von den Lungen-6 (KL-6) is a high molecular weight glycoprotein (>200 kDa) identified in 1985 [1]. KL-6 is expressed in various tissues, including the stomach, breast, pancreas, and lungs. Especially in the lungs, alveolar and bronchiolar epithelial cells express KL-6 [2]. KL-6 is released into the blood when these cells are proliferating, stimulated, or injured [3]. This is observed in patients with severe COVID-19, where the median serum KL-6 level reaches approximately 900 U/mL within 30 days and gradually decreases over the following 100 days [4]. Once released, KL-6 promotes the proliferation and survival of lung fibroblasts [2]. KL-6 is increased in lung, pancreatic, and breast cancers, as well as interstitial lung disease [2]. Interstitial lung disease has been extensively studied in relation to KL-6 as it may serve as a diagnostic marker or aid in evaluating disease activity and prognosis [3].

Given the potential utility of KL-6 in clinical diagnostics, it is necessary to establish a valid reference interval. Reference intervals, analytical performance, and biological variation are essential for interpreting laboratory results. Clinicians assess these parameters when determining whether a test result is normal or abnormal. Thus, establishing reference intervals in healthy individuals is crucial to determine optimal clinical limits for diagnosis and therapy guidance.

Only a few studies have investigated the reference intervals for KL-6. A Japanese study determined reference intervals for serum KL-6 using an enzyme immunoassay [5]. Another study determined the median serum KL-6 values in healthy controls [6]. To our knowledge, no study has investigated the reference intervals for plasma KL-6 in a population of Northern European descent using a fully automated latex-particle-enhanced turbidimetric immunoassay (LETIA). In order to evaluate the potential role of KL-6 as a biomarker in this population, it is necessary to establish reference intervals. Therefore, the objective of the present study is to establish reference intervals for plasma KL-6 according to the Clinical and Laboratory Standards Institute (CLSI) guideline EP28-A3c [7].

## 2. Materials and Methods

We employed the direct sampling technique recommended by the CLSI guideline EP28-A3c, where individuals were selected a priori. The reference subjects were blood donors from the Danish Blood Services at Biochemistry and Immunology, Lillebaelt Hospital, University Hospital of Southern Denmark, Denmark. In total, 243 residual samples were collected. After excluding two samples that failed during analysis and one outlier, the study population comprised 240 individuals. Blood donors in the Region of Southern Denmark are volunteers aged 17–70 years with a mean age of 43 years. To be eligible as blood donors, individuals must be healthy, weigh at least 50 kg, and meet specific criteria outlined in the Standards for Transfusion Medicine. These criteria include not having conditions such as symptomatic or systemically treated asthma, chronic obstructive pulmonary disease, asbestosis, tuberculosis, infectious diseases, cancer, anemia, blood disorders, thrombosis, severe autoimmune disorders, heart disease, and kidney disease; no recent surgery; no sexual risk behavior; no drug abuse; no blood transfusion; no pregnancy; no lactation; and more. Additionally, donors are excluded if they use medication incompatible with blood donation [8]. Immediately before blood donation, the participants answered a questionnaire about their health, medication use, and travel history, which may exclude them from donating.

Plasma samples were collected from 1 April to 30 June 2020. Blood samples were drawn from the antecubital vein into BD Vacutainer lithium heparin tubes (Becton Dickinson, Franklin Lakes, NJ, USA). Immediately after venipuncture, the samples were centrifuged at 2654 g for five minutes using an automated Hettich centrifuge (Andreas Hettich GmbH & Co. KG, Tuttlingen, Germany). The plasma was transferred to new tubes and frozen at −80 °C until analysis.

KL-6 was measured using an LETIA with the Nanopia KL-6 reagent (Sekisui Medical, Tokyo, Japan) according to the manufacturer’s protocol [9]. Plasma samples were analyzed in December 2021 in an open channel on a cobas 8000 module c502 (F. Hoffmann-La Roche Ltd., Basel, Switzerland). According to the manufacturer, the largest coefficient of variation for KL-6 is 1.4% at a mean level of 373 U/mL, and the method demonstrated linearity between 50 and 5000 U/mL.

The results were tested for normality using a Q-Q plot and Shapiro–Wilk’s test. A Wilcoxon–Mann–Whitney test was performed to compare females and males. An outlier check was conducted using Dixon’s method [10]. Reference intervals for KL-6 were determined using a parametric quantile approach for the common population and for females and males separately. The reference intervals corresponded to the 2.5th to 97.5th percentile (central 95%). The need for sex partitioning was evaluated based on the recommendations of Lahti et al. [11]. After estimating the common reference interval, the proportions of the two subgroups outside the reference interval were assessed. Subgroup-specific reference intervals were calculated if the proportions exceeded 4.1% or fell below 0.9%. Similar statistical methods were applied to analyze secondary data from Berastegui et al. [6]. A detailed description of these analyses can be found in the Supplementary Materials.

Statistical analyses were conducted using Microsoft Excel (2016) with the Analyse-it add-in for Microsoft Excel (version 2.30). The density plot was produced using the ggplot2 package version 3.4.2 for R 4.1.1 (The R Foundation for Statistical Computing, Vienna, Austria).

## 3. Results

The study population comprised 240 healthy blood donors. Table 1 shows a summary of the plasma KL-6 values according to sex.

### 3.1. Distribution

**Figure 1:**
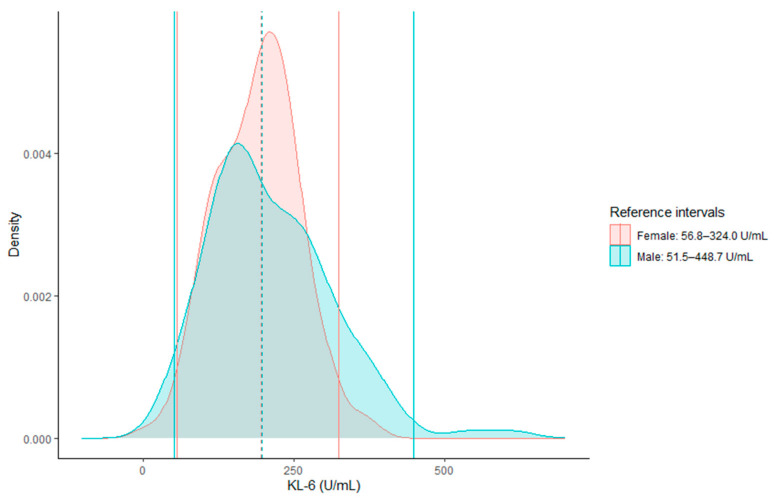
Distribution of plasma Krebs von den Lungen-6 (KL-6) levels in 240 blood donors according to sex. The red and blue lines represent females and males, respectively. The dotted and solid lines represent the medians and the reference intervals, respectively.

The common population’s mean (SD) value was 201.5 (87.1). In females, the mean (SD) value was 190.4 (68.0) and in males, it was 212.7 (102.0). Figure 1 shows the distribution of KL-6 values according to sex. In the overall population, the kurtosis and skewness were 2.13 and 0.9, respectively, indicating a leptokurtic and right-skewed distribution. A significant Shapiro–Wilk test confirmed a non-Gaussian distribution (*p* < 0.001). The distribution of results among females followed a Gaussian distribution, while that of males did not.

### 3.2. Sex Differences

Females and males displayed similar KL-6 values with median values of 195.5 and 197.7, respectively (*p* = 0.225). Among females, 1.6% fell below the lower limit of the common reference interval, while 0.0% were above the upper limit. For males, 4.2% fell below the lower limit and 3.3% exceeded the upper limit. As less than 0.9% of the female results were above the common reference interval and more than 4.1% of the male results were below the common reference interval, sex partitioning was necessary.

### 3.3. Reference Intervals

After exponential transformation, the kurtosis and skewness were 0.03 and 0.0, respectively, suggesting closeness to a normal distribution. This was confirmed by a Shapiro–Wilk test (*p* = 0.984). Using a parametric quantile approach on the transformed data, the common reference interval for KL-6 was determined to be 59.4–398.5 U/mL (95% confidence intervals (CI) for the lower and upper limits were 47.3–71.9 and 369.5–430.1, respectively). The reference interval for females was 56.8–324.0 U/mL (95% CIs for the lower and upper limit were 36.1–77.6 and 303.3–344.7, respectively) and for males, it was 51.5–448.7 U/mL (95% CIs for the lower and upper limits were 32.8–71.2 and 397.3–508.1, respectively).

### 3.4. Secondary Data Analysis

Using data from the study conducted by Berastegui et al., the common reference interval for KL-6 was determined to be 120.0–566.7 U/mL (95% CIs for the lower and upper limits were 101.6–139.7 and 485.9–680.8, respectively). The reference interval for females was 95.3–432.6 U/mL (95% CIs for the lower and upper limits were 54.8–135.9 and 392.1–473.2, respectively) and for males, it was 114.5–677.1 U/mL (95% CIs for the lower and upper limits were 86.8–145.3 and 516.3–1094.3, respectively). A detailed description of these data can be found in the Supplementary Materials.

## 4. Discussion

In this study, we determined the reference intervals for plasma KL-6 using an LETIA. We found it necessary to establish separate reference intervals for females and males.

Our study found that the upper reference limit for males is significantly higher than for females, as the confidence intervals do not overlap. A study conducted by Kobayashi et al. used an enzyme immunoassay to determine the serum KL-6 reference interval in 273 Japanese individuals, which ranged from 94.9 to 458.2 U/mL [5]. Similar to our study, they observed higher KL-6 levels in males than in females. A study by Berastegui et al. estimated the median serum KL-6 value as 265.8 U/mL in 100 Spanish individuals using an enzyme-linked immunosorbent assay (ELISA). The study found no difference between females and males when comparing medians [6]. However, even with smaller differences between medians, previous studies have showed that without partitioning, the proportions of a subclass outside the reference interval might be very different from the desired 2.5% on each side [7]. Similar to our study, the distribution of results in the Spanish female population followed a Gaussian distribution, while it did not in the male population. Biomarker distributions are often complex because of natural heterogeneity; thus, the different distributions in females and males support the need for partitioned reference intervals.

The Japanese and Spanish studies determined normal serum KL-6 levels in healthy individuals using enzyme immunoassays, which may explain any bias between the studies. A Korean study compared the KL-6 LETIA and ELISA methods according to the CLSI EP9-A3 guideline and found a strong correlation between the two methods. The ELISA showed lower serum KL-6 concentrations than the LETIA, with an average bias of −15% [12]. In the Japanese and Spanish populations, the lower and upper limits are higher compared to our study (see Table S2 in the Supplementary Materials). This may be attributed to the use of different matrices. Previous studies analyzing plasma KL-6 using ELISA reported similar medians to our study in healthy controls [13,14,15]. Further investigations determining KL-6’s stability in plasma and serum are required to confirm any differences between matrices. Additionally, population characteristics, such as smoking, comorbidities, and medication, may also play a role, as well as inter-assay disagreement [16,17]. When comparing studies, caution should be exercised if different assays and matrices are used. Nonetheless, it emphasizes the importance of determining reference intervals in various populations using all relevant assays and matrices and providing detailed descriptions of the parameters used.

The strengths of our study include the use of the commercially available Nanopia KL-6 reagent, recognized for automated use on the cobas 8000 platform, and that it is easily implemented in laboratory routines. The analyses were conducted as batch analyses by trained laboratory technicians at an accredited laboratory.

The limitations of the present study include the lack of an external quality program for KL-6, which makes reference interval transfer between laboratories challenging. Moreover, there were limited donor data other than sex and the general eligibility requirements. The inclusion criteria, exclusion criteria, and pre-donation questionnaire for blood donors are standardized nationally, so we expect the blood donors in our study to be representative of Danish blood donors in general. A subpopulation included in the Danish Blood Donor Study Genomic Cohort had a mean age of 41 years, which is comparable to the mean age in the present study [18]. The age prevalence of Danish blood donors remains constant between 25 and 55 years but differs outside this interval. Other sociodemographic factors may vary across the donor population. In Denmark, blood donation is underrepresented in minority ethnic groups, low-income groups, high-income groups, males living without a partner, and women with children [19]. Hence, caution should be taken when assessing KL-6 levels at an individual level.

Regarding comorbidities, Cho et al. found no significant differences between healthy and disease controls who suffered from infectious lung disease, chronic obstructive pulmonary disorder, or radiation-induced pulmonary fibrosis [12]. Additionally, Edgren et al. suggested that implementing exclusion criteria for Scandinavian blood donors ensures a healthy donor population. Their study found significantly lower mortality among blood donors than the background population and a reduced risk for smoking-related cancers [20]. Hence, comorbidities and smoking may have a lower impact on mortality and cancer among Danish blood donors. In the Danish Blood Donor Study Genomic Cohort, 12% of participants were smokers, which may be comparable to the prevalence in the present study [18]. However, there is no significant difference in KL-6 levels between smokers and non-smokers [5].

An increasing number of scientific reports are being published on KL-6, and its clinical utility as a prognostic and diagnostic biomarker is becoming increasingly evident [3]. Radiological and pulmonary function tests are the only diagnostic and prognostic alternatives for interstitial lung disease. However, these tests are time consuming, expensive, and generally challenging to quantitate compared to blood testing. Performing the LETIA requires only 10 min, while an ELISA takes hours. Nevertheless, methodological differences must be considered when interpreting laboratory results. This study provides the necessary reference intervals to utilize the KL-6 LETIA in everyday clinical practice. In future studies, it may be relevant to investigate KL-6 reference intervals using the LETIA in a larger setting, considering different age groups and ethnic populations to achieve better representativeness.

In conclusion, the present study demonstrates the necessity of sex partitioning when assessing KL-6 reference intervals. We established these reference intervals, which provide a solid evidence base for future studies involving KL-6.

## Figures and Tables

**Table 1:** Plasma Krebs von den Lungen-6 (KL-6) results in 240 blood donors.

	*N*	Median	Interquartile Range	Min–Max
Common	240	196.1 U/mL	138.8–250.5 U/mL	8.5–613.4 U/mL
Female	121	195.5 U/mL	137.3–239.2 U/mL	8.5–376.9 U/mL
Male	119	197.7 U/mL	140.1–274.8 U/mL	30.8–613.4 U/mL

## Data Availability

The data presented in this study are available in Supplementary Materials.

## Supplementary Materials

The following supporting information can be downloaded at: https://www.mdpi.com/article/10.3390/diagnostics13111951/s1, Secondary data analysis; Table S1: Krebs von den Lungen-6 (KL-6) results for the 100 healthy controls. Table S2: Reference intervals for Krebs von den Lungen-6 comparing existing literature to the present study.

## References

[1] Nobuoki K., Seishi K., Yukikazu A., Hirofumi F., Michio Y., Mitoshi A. (1989). New Serum Indicator of Interstitial Pneumonitis Activity: Sialylated Carbohydrate Antigen KL-6. Chest.

[2] Ishikawa N., Hattori N., Yokoyama A., Kohno N. (2012). Utility of KL-6/MUC1 in the clinical management of interstitial lung diseases. Respir. Investig..

[3] Zhang H., Chen L., Wu L., Huang J., Li H., Wang X., Weng H. (2020). Diagnostic and prognostic predictive values of circulating KL-6 for interstitial lung disease: A PRISMA-compliant systematic review and meta-analysis. Medicine.

[4] Deng K., Fan Q., Yang Y., Deng X., He R., Tan Y., Lan Y., Deng X., Pan Y., Wang Y. (2021). Prognostic roles of KL-6 in disease severity and lung injury in COVID-19 patients: A longitudinal retrospective analysis. J. Med. Virol..

[5] Kobayashi J., Itoh Y., Kitamura S., Kawai T. (1996). Establishment of reference intervals and cut-off value by an enzyme immunoassay for KL-6 antigen, a new marker for interstitial pneumonia. Rinsho Byori.

[6] Berastegui C., Gómez-Ollés S., Mendoza-Valderrey A., Pereira-Veiga T., Culebras M., Monforte V., Saez B., López-Meseguer M., Sintes-Permanyer H., Ruiz de Miguel V. (2020). Use of serum KL-6 level for detecting patients with restrictive allograft syndrome after lung transplantation. PLoS ONE.

[7] CLSI (2008). Defining, Establishing, and Verifying Reference Intervals in the Clinical Laboratory; Approved Guideline—Third Edition.

[8] Dansk Selskab for Klinisk Immunologi (2022). Transfusionsmedicinske Standarder.

[9] Sekisui Medical Nanopia KL-6 Reagent. https://www.sekisuimedical.jp/english/business/diagnostics/biochemistry/n_kl/pdf/Nanopia%20KL-6Reagent.pdf.

[10] Dixon W.J. (1953). Processing Data for Outliers. Biometrics.

[11] Lahti A., Petersen P.H., Boyd J.C., Rustad P., Laake P., Solberg H.E. (2004). Partitioning of nongaussian-distributed biochemical reference data into subgroups. Clin. Chem..

[12] Cho E.J., Park K.J., Ko D.H., Koo H.J., Lee S.M., Song J.W., Lee W., Lee H.K., Do K.H., Chun S. (2019). Analytical and Clinical Performance of the Nanopia Krebs von den Lungen 6 Assay in Korean Patients With Interstitial Lung Diseases. Ann. Lab. Med..

[13] Sato H., Callister M.E.J., Mumby S., Quinlan G.J., Welsh K.I., duBois R.M., Evans T.W. (2004). KL-6 levels are elevated in plasma from patients with acute respiratory distress syndrome. Eur. Respir. J..

[14] Ishizaka A., Matsuda T., Albertine K.H., Koh H., Tasaka S., Hasegawa N., Kohno N., Kotani T., Morisaki H., Takeda J. (2004). Elevation of KL-6, a lung epithelial cell marker, in plasma and epithelial lining fluid in acute respiratory distress syndrome. Am. J. Physiol. Lung Cell. Mol. Physiol..

[15] Nathani N., Perkins G.D., Tunnicliffe W., Murphy N., Manji M., Thickett D.R. (2008). Kerbs von Lungren 6 antigen is a marker of alveolar inflammation but not of infection in patients with acute respiratory distress syndrome. Crit. Care.

[16] Song M.A., Seffernick A.E., Archer K.J., Mori K.M., Park S.Y., Chang L., Ernst T., Tiirikainen M., Peplowska K., Wilkens L.R. (2021). Race/ethnicity-associated blood DNA methylation differences between Japanese and European American women: An exploratory study. Clin. Epigenet..

[17] Aloisio E., Braga F., Puricelli C., Panteghini M. (2021). Prognostic role of Krebs von den Lungen-6 (KL-6) measurement in idiopathic pulmonary fibrosis: A systematic review and meta-analysis. Clin. Chem. Lab. Med..

[18] Hansen T.F., Banasik K., Erikstrup C., Pedersen O.B., Westergaard D., Chmura P.J., Nielsen K., Thørner L., Hjalgrim H., Paarup H. (2019). DBDS Genomic Cohort, a prospective and comprehensive resource for integrative and temporal analysis of genetic, environmental and lifestyle factors affecting health of blood donors. BMJ Open.

[19] Burgdorf K.S., Simonsen J., Sundby A., Rostgaard K., Pedersen O.B., Sørensen E., Nielsen K.R., Bruun M.T., Frisch M., Edgren G. (2017). Socio-demographic characteristics of Danish blood donors. PLoS ONE.

[20] Edgren G., Tran T.N., Hjalgrim H., Rostgaard K., Shanwell A., Titlestad K., Wikman A., Norda R., Jersild C., Wideroff L. (2007). Improving health profile of blood donors as a consequence of transfusion safety efforts. Transfusion.

