# Supplementary material for: Establishing Sex-Dependent Reference Intervals for KL-6 in Danish Adults
Source: Diagnostics (Basel). 2023 Jun 2;13(11):1951. doi: 10.3390/diagnostics13111951 (PMC10253194; doi:10.3390/diagnostics13111951)
Supplement: Supplementary file 1 [file diagnostics-13-01951-s001.zip › diagnostics-2389539-supplementary.docx]

# Supplementary materials

## Secondary data analysis

### Methods

Secondary data from healthy controls were obtained from Berastegui C, Gómez-Ollés S, Mendoza-Valderrey A, et al. 2020. S1 Table: Metadata in a cross-sectional lung transplanted cohort to study KL-6 levels; <https://doi.org/10.1371/journal.pone.0226488.s001>. In total, data from 100 healthy controls were available, including 50 females and 50 males. The blood samples were collected between November 2016 and February 2017 from the blood bank “Banc de Sang i Teixits, BST”. KL-6 was measured in serum using the ELISA kit for KL-6 (Eida Co. Ltd., Tokyo, Japan) according to the manufacturer’s protocol.

The secondary data were tested for normality using a qq-plot and Shapiro-Wilk’s test. A Wilcoxon-Mann-Whitney test was performed comparing females and males. Outlier-check was done and no outliers were identified. Reference intervals for KL-6 were determined using a parametric quantile approach in both the common, female, and male populations. The reference intervals corresponded to the 2.5th to 97.5th percentile (central 95%). The need for sex partitioning was assessed. After estimating the common reference interval, an assessment of the proportions of the two subgroups outside the reference interval was done. Subgroup-specific reference intervals were calculated if the proportions exceeded 4.1% or fell below 0.9%.

Statistical analyses were conducted in Microsoft Excel (2016) using Analyse-it for Microsoft Excel (version 2.30).

### Results

The secondary data population comprised 100 healthy controls. A summary of the serum KL-6 values according to sex are shown in Table S1.

**Table S1:** Krebs von den Lungen-6 (KL-6) results for the 100 healthy controls.

|  | **N** | **Median** | **Interquartile range** | **Min-max** |
| --- | --- | --- | --- | --- |
| Common | 100 | 265.8 U/mL | 199.3-345.8 U/mL | 102.7-927.3 U/mL |
| Female | 50 | 253.8 U/mL | 198.2-317.7 U/mL | 129.9-459.8 U/mL |
| Male | 50 | 273.2 U/mL | 207.1-386.9 U/mL | 102.7-927.3 U/mL |

The mean (SD) values were 282.8 (119.6) combined, 264.0 (85.6) in females, and 301.7 (144.4) in males. In the common population, kurtosis and skewness were 7.82 and 2.0, respectively, suggesting a leptokurtic and right-skewed distribution. A significant Shapiro-Wilk test confirmed a non-Gaussian distribution (p < 0.001). The female results followed a Gaussian distribution, while the male results did not.

Females and males displayed comparable KL-6 values with medians of 253.8 and 273.2, respectively (p = 0.278). The female proportion below the lower limit of the common reference interval was 2.0%, while 4.0% was above the upper limit. For males, 0.0% was below the lower limit and 0.0% above the upper limit. Because less than 0.9% of the male results were below and above the common reference interval, sex partitioning was necessary.

After exponential transformation, kurtosis and skewness were -0.51 and 0.1, suggesting closeness to a normal distribution, which was confirmed by a Shapiro-Wilk test (p = 0.479). Using a parametric quantile approach on the transformed data, the common reference interval for KL-6 was determined to be 120.0-566.7 U/mL (95% confidence intervals (CIs) for the lower and upper limit were 101.6-139.7 and 485.9-680.8, respectively). The reference interval for females was 95.3-432.6 U/mL (95% CIs 54.8-135.9 and 392.1-473.2, respectively) and for males 114.5-677.1 U/mL (95% CIs 86.8-145.3 and 516.3-1094.3, respectively).

## Comparative table

**Table S2:** Reference intervals for Krebs von den Lungen-6 comparing existing literature to the present study.

|  | **Present study** | | **Berastegui et al.** | | **Kobayashi et al.** | |
| --- | --- | --- | --- | --- | --- | --- |
|  | **N** | **Reference interval** | **N** | **Reference interval** | **N** | **Reference interval** |
| Common | 240 | 59.4-398.5 U/mL | 100 | 120.0-566.7 U/mL | 273 | 94.9-458.2 U/mL |
| Female | 121 | 56.8-324.0 U/mL | 50 | 95.3-432.6 U/mL | 153 | 92.0-435.6 U/mL |
| Male | 119 | 51.5-448.7 U/mL | 50 | 114.5-677.1 U/mL | 120 | 104.0-492.5 U/mL |
